# Increased Clinical Signs and Mortality in IFNAR^(−/−)^ Mice Immunised with the Bluetongue Virus Outer-Capsid Proteins VP2 or VP5, after Challenge with an Attenuated Heterologous Serotype

**DOI:** 10.3390/pathogens12040602

**Published:** 2023-04-15

**Authors:** Houssam Attoui, Fauziah Mohd Jaafar, Baptiste Monsion, Bernard Klonjkowski, Elizabeth Reid, Petra C. Fay, Keith Saunders, George Lomonossoff, David Haig, Peter P. C. Mertens

**Affiliations:** 1 UMR1161 VIROLOGIE, INRAE, Ecole Nationale Vétérinaire d’Alfort, ANSES, Université Paris-Est, F-94700 Maisons-Alfort, France; faojaafar@gmail.com (F.M.J.); baptiste.monsion@vet-alfort.fr (B.M.); bernard.klonjkowski@vet-alfort.fr (B.K.); 2 One Virology, The Wolfson Centre for Global Virus Research, Sutton Bonington Campus, School of Veterinary Medicine and Science, University of Nottingham, Leicestershire LE12 5RD, UK; elizabeth.reid1@nottingham.ac.uk (E.R.); p.fay@wellcome.org (P.C.F.); david.haig1@nottingham.ac.uk (D.H.); peter.mertens@nottingham.ac.uk (P.P.C.M.); 3 John Innes Centre, Department of Biochemistry and Metabolism, Norwich NR4 7UH, UK; keith.saunders@jic.ac.uk (K.S.); george.lomonossoff@jic.ac.uk (G.L.)

**Keywords:** bluetongue virus (BTV), vaccination, VP2, VP5, heterologous serotype challenge

## Abstract

Bluetongue is an economically important disease of domesticated and wild ruminants caused by bluetongue virus (BTV). There are at least 36 different serotypes of BTV (the identity of which is determined by its outer-capsid protein VP2), most of which are transmitted by *Culicoides* biting midges. IFNAR^(−/−)^ mice immunised with plant-expressed outer-capsid protein VP2 (rVP2) of BTV serotypes -1, -4 or -8, or the smaller outer-capsid protein rVP5 of BTV-10, or mock-immunised with PBS, were subsequently challenged with virulent strains of BTV-4 or BTV-8, or with an attenuated clone of BTV-1 (BTV-1RG_C7_). The mice that had received rVP2 generated a protective immune response against the homologous BTV serotype, reducing viraemia (as detected by qRT-PCR), the severity of clinical signs and mortality levels. No cross-serotype protection was observed after challenge with the heterologous BTV serotypes. However, the severity of clinical signs, viraemia and fatality levels after challenge with the attenuated strain of BTV-1 were all increased in mice immunised with rVP2 of BTV-4 and BTV-8, or with rVP5 of BTV10. The possibility is discussed that non-neutralising antibodies, reflecting serological relationships between the outer-capsid proteins of these different BTV serotypes, could lead to ‘antibody-dependent enhancement of infection’ (ADE). Such interactions could affect the epidemiology and emergence of different BTV strains in the field and would therefore be relevant to the design and implementation of vaccination campaigns.

## 1. Introduction

Virus neutralisation can occur when antibodies bind to ‘neutralisation epitopes’ on cell-attachment proteins on the virus particle surface. This blocks interactions with virus receptors on host cells, preventing the initiation of infection and blocking subsequent virus replication [1]. Not all antibodies that bind to the virus surface cause neutralisation, although non-neutralising antibodies can still cause aggregation of virus particles, leading to their clearance by cellular components of the host’s immune system.

Viral vaccines are prophylactic therapeutic formulations intended to fully or partially protect the vaccinee against virus infections and/or severe clinical signs of infection. Most vaccines achieve these goals by inducing the production of neutralising antibodies and/or by promoting T-cell responses that can destroy cells that do become infected. Vaccines that induce protective cell-mediated immunity (CMI—an important component of acquired immunity) are mainly viral-vector based, targeting pathogen-specific antigens synthesised *in cellulo* [2,3].

Most vaccines that have been approved as prophylactic measures for diseases caused by pathogens (parasites, bacteria or viruses) stimulate a protective humoral response [4]. However, infected or vaccinated animals that have antibodies against a single serotype of a multi-serotype virus can show distinct or even more severe clinical signs after infection with a different serotype [5,6]. The mechanisms involved are collectively described as ‘antibody-dependent enhancement of infection’ (ADE) and are seen predominantly during infections by RNA viruses with multiple serotypes. They include an antibody-mediated enhancement of infectivity [7] caused by the Fc region of antibodies bound to the virus particle interacting with Fc receptors on the macrophage surface, facilitating particle uptake and increasing their infectivity for these cells (as seen with dengue virus (DENV) and feline infectious peritonitis virus (FIPV) infections [5,6,8]). A prerequisite for this ADE mechanism is that the virus is able to infect and replicate in macrophages and other FcR+ cells of the reticuloendothelial system.

ADE can also be caused by virus particle surface interactions with non-neutralising antibodies resulting in excessive formation of immune complexes, leading to cell-mediated immunopathology and/or induction of cytokine cascades [9,10,11]. Immune complexes and secretion of pro-inflammatory cytokines can induce immune cell recruitment and activation of the complement cascade. Such immune enhancement is best known to occur during infections by respiratory viruses, leading to enhanced respiratory disease (ERD). Viruses which can cause ERD include respiratory syncytial (RS) and measles virus, particularly in subjects that were previously vaccinated with ‘killed virus’ vaccines [9,12,13,14,15]. ADE/ERD were observed in both SARS-CoV and MERS-CoV infections, *in vitro* and *in vivo* [9]. It has been suggested that immune complex formation, complement deposition and local immune activation are likely underlying mechanisms of ADE in COVID-19 immunopathology [9].

Many *Orbivirus* species (including *Bluetongue virus* (BTV), *Epizootic haemorrhagic disease virus* (EHDV) and *African horse sickness virus* (AHSV)) exist as multiple virus serotypes. The identities of individual orbivirus serotypes are controlled by the specificity of interactions between the viral cell attachment protein (major outer-capsid protein (OC1)) and neutralising antibodies, which can be analysed in neutralisation assays [16]. In the midge-borne orbiviruses (which includes BTV), the amino acid (aa) sequence of the larger outer-capsid protein VP2 (OC1) therefore determines the virus serotype [17]. There are currently more than 36 recognised serotypes of BTV, which we speculate could lead to ADE in areas where multiple serotypes are in circulation. The orbivirus outer capsid also contains a second protein, VP5 (OC2), which mediates membrane penetration during the initiation of cellular infection [18]. Although the VP5 of BTV may contribute to the determination of the virus serotype [19,20,21,22] (possibly via interactions with VP2), it does not appear to bind neutralising antibodies [23].

After the initial infection of a ruminant host, usually via the bite of an infected adult *Culicoides* [24], dendritic cells and macrophages deliver infectious BTV particles from the skin to the lymph nodes, where replication occurs and the virus is subsequently disseminated to other organs [25,26,27]. Replication in mononuclear phagocytes and vascular endothelial cells results in an enhanced production and release of cytokines and other vasoactive mediators, increasing fever in infected ruminants [26]. This can lead to vascular injury, exudation of fluid from blood vessels and haemorrhages in many tissues (including the lungs), contributing to respiratory disease, which is a frequent clinical feature of severe BTV infections [10,11,27].

The level of viraemia in an infected host reflects the replication of BTV and release/circulation of the virus into the host’s blood stream but does not show a fixed correlation with the severity of clinical signs. Cattle infected with BTV can often be largely asymptomatic after infection, although they frequently show a higher viraemia than infected sheep which often show more severe clinical signs of the disease [28]. In sheep and cattle, BTV viraemia can also persist for long periods post-infection, even though clinical signs of disease have subsided [29,30]. However, an early and rapid rise in viraemia post-infection may be an early indicator of the severity of disease outcomes within an individual host.

We have been studying the pathogenesis and control of BTV infections using plant expressed outer-capsid proteins and challenge strains of BTV serotypes -1, -4 and -8, which have caused recent disease outbreaks in Europe and have been used in associated vaccination programmes [10,27,31]. In previous studies, we showed that the plant-expressed rVP2 proteins of BTV-4 and BTV-8 (as described here) raised antibodies in IFNAR^(−/−)^ mice and rabbits which recognise the homologous rVP2 proteins and viruses in ELISA and serum neutralisation tests (SNTs) [32,33]. The immune responses generated in mice were also protective against lethal challenge with virulent strains of the homologous but not the heterologous serotype. After challenge with the heterologous serotype, some of the mice developed a higher early viraemia than the non-immunised animals, suggesting an ADE-type response. However, all of the unprotected mice (non-immunised, or immunised with an rVP2 of the heterologous serotype) died within 3 to 5 days post challenge, leaving insufficient time to fully assess the possibility of enhanced clinical signs of infection [32].

In this follow-up study, we have used virulent strains of BTV-4 and BTV-8, as well as an attenuated (non-lethal) strain of BTV-1, to challenge IFNAR^(−/−)^ mice, confirming the protective response against the homologous serotype after immunisation with plant-expressed rVP2 proteins. Longer survival times after challenge with the attenuated BTV-1 strain also provided an opportunity to observe any enhanced clinical signs of infection (or fatalities) in animals previously immunised with the plant-expressed rVP2 or rVP5 proteins of heterologous serotypes.

## 2. Materials and Methods

### 2.1. Ethics Statement

Animal immunisation was conducted in agreement with the European animal welfare legislation of the EU (Directive 2010/63/EU). Experimentation protocols were approved by the Ethics Committee for animal experimentation of Anses-EnvA-UPEC (project licence Number: 19-028).

### 2.2. Cell Cultures and Viruses

Baby hamster kidney BSR cells (a clone of BHK-21 cells [34]) were grown at 37 °C in Dulbecco’s modified Eagle medium (DMEM) supplemented with 10% foetal bovine serum and 100 IU of penicillin/100 µg of streptomycin per ml, in 5% CO_2_. *Culicoides sonorensis* KC cells were grown at 28 °C in Schneider’s insect medium supplemented as above.

The virus clone BTV-1RG_C7_ was generated by reverse genetics as described previously [35], based on the genome sequence of the BTV-1 reference strain BTV-1RSArrrr/01 (available online: https://www.reoviridae.org/dsRNA_virus_proteins/ReoID/btv-1.htm#RSArrrr/01, accessed on 19 December 2020). This attenuated virus infects IFNAR^(−/−)^ mice but causes only mild clinical signs, followed by a full recovery [35]. The strains of BTV-4RSArrrr/04 (available online: https://www.reoviridae.org/dsRNA_virus_proteins/ReoID/btv-4.htm#RSArrrr/04, accessed on 19 December 2020) and BTV8RSArrrr/08 (available online: https://www.reoviridae.org/dsRNA_virus_proteins/ReoID/btv-8.htm#RSArrrr/08, accessed on 19 December 2020) that were used were obtained from the orbivirus reference collection at the Pirbright Institute in the UK.

### 2.3. Infection of Cell Cultures and Purification of Viruses Prior to Inoculation into IFNAR^(−/−)^ Mice

Monolayers of BSR cells (85% confluence) were infected with BTV-1RG_C7_ or reference strains of either BTV-4 or BTV-8 at multiplicities of infection (MOIs) of 0.05 pfu/cell. Cells were then incubated at 37 °C for 4 days until full cytopathic effects (CPEs) were observed. Cells and debris were pelleted by centrifugation at 2000× *g* for 10 min at 4 °C. Cell pellets were suspended in culture medium without FBS and subjected to 10 strokes in a Dounce homogeniser before being treated with Vertel XF (Sigma) to free virus particles from interaction with cell debris. This semi purified virus was used to infect KC cells (MOI = 0.05) at 28 °C for 7 days. BTV from KC cell culture supernatants at 7 days post-infection was used in challenge experiments.

### 2.4. Experimental Design

Representative VP2 and VP5 aa sequences for serotypes 1 to 27 were aligned using ClustalX [36]. Phylogenetic analyses were performed using MegaX [37]. Aligned sequences were used to generate maximum-likelihood trees with the Dayhoff substitution model and the trees were inferred using the nearest-neighbour interchange. Phylogenetic groupings of VP2 or VP5 aa sequences helped inform the choice of the viral proteins from BTV strains in different groups to immunise IFNAR^(−/−)^ mice. Each mouse was immunised twice (day 0 and day 14) with 5 µg of plant-expressed rVP2 of BTV serotypes -1, -4 or -8, or 5 µg of plant-expressed VP5 (rVP5) of BTV-10, mixed with the adjuvant Montanide ISA50V (SEPPIC). The ratio of adjuvant to antigen solution was 1/1. Recombinant VP2 of BTV-1 (accession number KP821004), BTV-4 (accession number KP821064) or BTV-8 (accession number KP821074) were produced in *Nicotiana benthamiana* and purified as previously described [32,33]. Recombinant VP5 of BTV-10 was produced in *Nicotiana benthamiana* and purified as previously described [38].

Fifteen groups of IFNAR^(−/−)^ mice, each containing five 8–10 weeks old mice, were used in this study (Table 1). The mice were challenged with different BTV strains (as indicated in Table 1) 14 days after the second immunisation. On the day of challenge, each mouse was injected with a dose of 10^3^ pfu of live BTV and all mice were observed for 14 days post-challenge.

The statistical significance of differences in the real-time RT-PCR results between groups of mice was assessed using an analysis of variance (ANOVA), which was carried out using Tukey’s test (differences are considered as statistically significant when *p* < 0.05: confidence interval of 95%) with HSD (honestly significant difference). Detailed results are given in Tables S3–S5.

### 2.5. Blood Collection, RNA Extraction and Real-Time RT-PCR

Approximately 50 µL of blood was collected from the retro-orbital sinus of each mouse on days 0, 5, 7 and 12 post-challenge and transferred into EDTA-containing tubes. RNA was extracted from these samples using TRIzol reagent (Thermo Fisher, les Ulis, France). Briefly, 20 µL of blood was added to 1 mL of TRIzol and agitated vigorously for 30 s, followed by the addition of 200 µL of chloroform. RNA extractions were performed in duplicates. The tubes were agitated for 30 s and incubated on ice for 10 min to allow phase separation. After centrifugation at 12,000× *g* for 10 min, the supernatant was collected and the RNA was purified using the RNeasy minikit (Qiagen). The supernatant was mixed with 350 µL of absolute ethanol, transferred to the RNeasy column and further processed as described by the manufacturer. The RNA was eluted from the column in 40 µL of RNase-free water and real-time RT-PCR was performed as described earlier using primers previously described for the BTV genome segment 10 [32,39,40,41].

## 3. Results

### 3.1. Phylogenetic Analysis and Group Design

**Figure 1:**
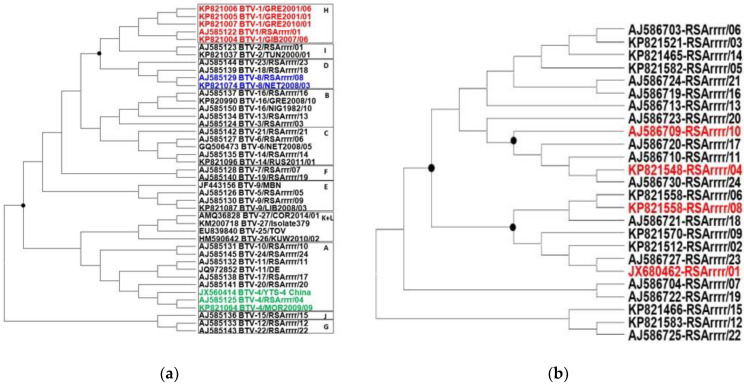
Maximum-likelihood trees constructed using representative BTV aa sequences, including all 24 reference strains of BTV-1 to BTV-24 (accession numbers are shown as part of strain designation). (**a**): VP2 aa sequence tree, which depicts previously defined nucleotypes A-L [17]; (**b**): VP5 aa sequence tree. Sequence alignment was generated by ClustalX and the tree was constructed with the help of the MegaX software using the Dayhoff substitution model/nearest-neighbour interchange. The serotypes used in the current study are indicated by coloured text.

VP2 is the most variable of the BTV proteins [17]. Phylogenetic analyses of VP2 aa sequences from the BTV serotypes selected for the study described here show identities ranging between 52% and 54% for different strains of BTV-1 and BTV-8 (placing them in separate clusters: groups H and D, Figure 1). More distant relationships (ranging between 40% and 42% aa identities) were identified between the VP2 of these serotypes and that of BTV-4 (group A, Figure 1). VP5 is the second most variable of the BTV proteins [42]. Previous phylogenetic analyses have shown aa sequence identity levels ranging from 98.1 to 55.7% between VP5 proteins of different BTV serotypes [39,43,44,45]. VP5 from BTV-1 and rVP5 of BTV-10 (used to immunise mice in the study described here) share 78.3% aa identity, while those of BTV-4 and BTV-10 are 95.8% identical, those of BTV-8 and BTV-10 are 78.9% identical and those of BTV-4 and BTV-8 are 79.5% identical. The BTV strains used here were selected partly to represent distantly related serotypes.

### 3.2. Clinical Observations

**Figure 2:**
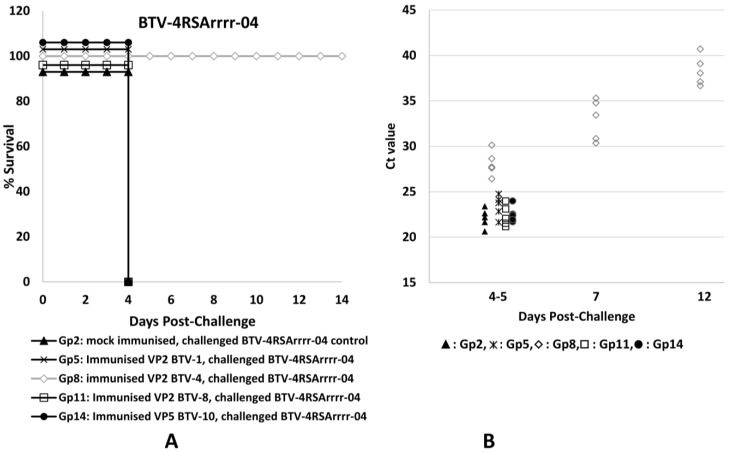
Survival curves (**A**) of mice challenged with BTV-4RSArrrr-04 and real-time RT-PCR CT values (**B**) of blood samples from individual mice in each of the five groups challenged with the same virus. Significant differences (*p* < 0.01) were observed between the group immunised with VP2 of BTV-4 and mice in all other groups, suggesting that VP2 of BTV-4 protects mice from a homologous challenge with BTV-4, hence mice in this group survived until the end of the experiment. No protection was observed in the heterologous challenge groups.

**Figure 3:**
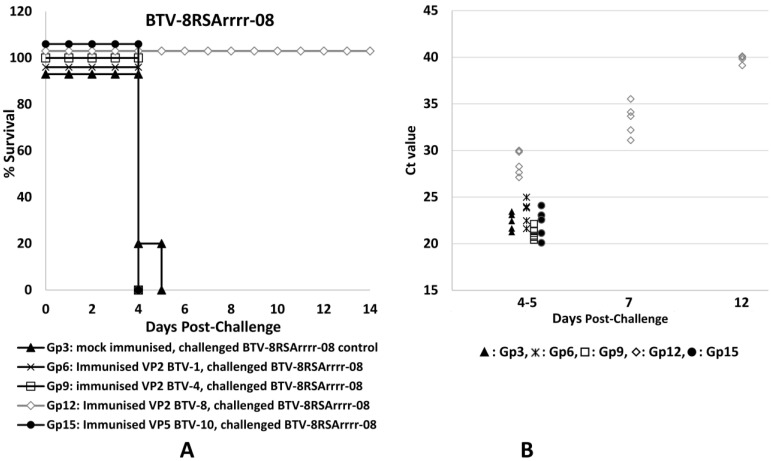
Survival curves (**A**) of mice challenged with BTV-8RSArrrr-08 and real-time RT-PCR CT values (**B**) of blood samples from individual mice in each of the five groups challenged with the same virus. Significant differences (*p* < 0.01) were observed between the group immunised with VP2 of BTV-8 and mice in all other groups, suggesting that VP2 of BTV-8 protects mice from a homologous challenge with BTV-8, hence mice in this group survived until the end of the experiment. No protection was observed in the heterologous challenge groups.

**Figure 4:**
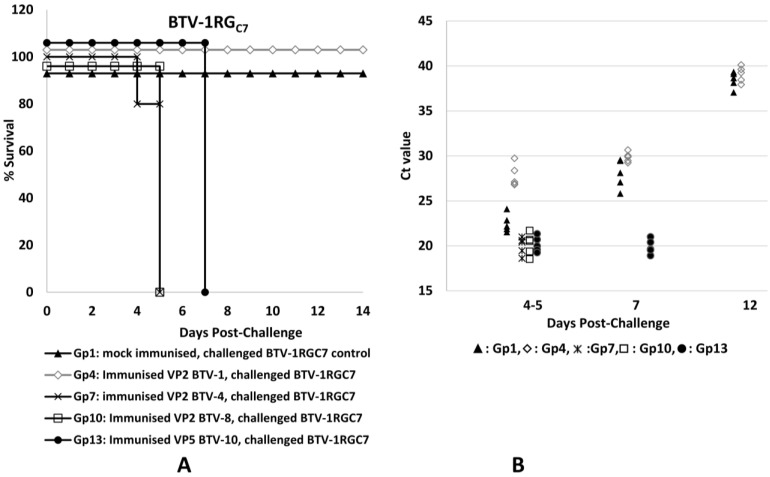
Survival curves (**A**) for mice challenged with BTV-1RG_C7_ and real-time RT-PCR CT values (**B**) of blood samples from individual mice in each of the five groups challenged with the same virus. Mock-immunised mice challenged with the virus developed mild clinical signs and recovered, surviving until the end of the experiment. Mice immunised with VP2 of BTV-1 and then challenged with BTV-1RG_C7_ were fully protected (*p* < 0.01), reflecting a serotype-specific neutralising response. Significant differences (*p* < 0.05) were observed between mock-immunised mice and mice immunised with VP2 of BTV-4 or BTV-8 and mice immunised with VP5 of BTV-10, which all developed more severe clinical sings than the mock-immunised mice and died between days 4 and 7 post-challenge.

The results of immunisation and challenge experiments in IFNAR^(−/−)^ mice are summarised in Table S1. Survival curves and Ct values for the levels of BTV RNA in blood samples (RNAemia) indicating the amount of virus present are shown in Figure 2, Figure 3 and Figure 4. The mice that were mock-immunised (with PBS) and then challenged with BTV-4 or BTV-8 (Figure 2 and Figure 3) all developed clinical signs typical of a severe BTV infection (as defined in Table S2), including lacrimation, scruffy hair and prostration, and all had died by day 5 post-challenge (p.c.). The mock-immunised mice challenged with the attenuated strain of BTV-1 developed mild clinical signs (as defined in Table S2), including lacrimation and scruffy fur, but made a full recovery, surviving to the end of the experiment on day 14 p.c. (Figure 4).

The mice immunised with plant-expressed rVP2 proteins of BTV-1, BTV-4 or BTV-8 and then challenged with the homologous BTV serotype were all protected, with no apparent clinical signs in those mice challenged with BTV-1 or BTV-4 (Table S1, groups 4 and 8, Figure 3 and Figure 4). Mice that received a homologous challenge with BTV-8 (group 12, Figure 2) did develop very mild clinical signs on days 4–5 p.c. but had made a full recovery by day 7 p.c. The mice immunised with rVP2 of BTV-1, BTV-4 or BTV-8 and then challenged with a heterologous serotype (BTV-4 or BTV-8, groups 9 and 11, Figure 2 and Figure 3) showed no indication of protection, developing severe clinical signs in a manner similar to the mock-immunised control animals challenged with the same viruses (groups 2 and 3). However, the mice immunised with rVP2 of BTV-4 or BTV-8 that received a heterologous challenge with the attenuated BTV-1RG_C7_ (groups 7 and 10, Figure 4) developed more severe clinical signs than the mock-immunised control group infected with the same virus (group 1), and had all died by day 5 p.c.

The mice immunised with VP5 of BTV-10 and then challenged with BTV-4 or BTV-8 (groups 14 and 15, Figure 2 and Figure 3) also showed severe clinical signs similar to those shown by the mock-immunised control groups challenged with these viruses (groups 2 and 3, Figure 2 and Figure 3), with all animals dying on day 4 p.c. Mice immunised with rVP5 of BTV-10 and then challenged with the attenuated BTV-1 RG_C7_ strain (group 13, Figure 4) all showed mild to severe clinical signs on day 5 p.c., but (unlike the mock-immunised group challenged with the same virus) these progressed to become much more severe, with all mice dying on day 7 p.c. The mice in this group therefore survived slightly longer than mice challenged with the same BTV-1RG_C7_ virus after immunisation with rVP2 of BTV-4 or BTV-8 (groups 7 and 10).

### 3.3. Real-Time PCR Assessment of BTV RNA in Mouse Blood (RNAemia)

CT values obtained by real-time RT-PCR (Table S1 and Figure 2, Figure 3 and Figure 4) showed that mice in the mock-immunised groups had similar levels of RNAemia, indicating higher levels of the virus in blood samples on days 4–5 post-infection with BTV-1 RG_C7_, BTV-4 or BTV-8 (CT values ranging between 20.63 and 23.45, with average CT values of 22.11 to 22.7). The mild clinical signs observed after challenge of the mock-immunised mice with BTV-1 RG_C7_ confirm that there is not a direct correlation between the severity of clinical signs and the level of viraemia, agreeing with reports of cattle vaccination/challenge studies [45].

Mice immunised with the rVP2s were protected against challenge with the homologous virus serotype, with higher mean CT values: 27.78 for BTV-1, 28.11 for BTV-4 and 28.6 for BTV-8 in these animals. This represents 5.6 to 6.2 CTs more than the corresponding mock-immunised groups of mice, showing that immunisation with the homologous proteins significantly reduces RNAemia post challenge (by ~50–63 fold). The protected mice all survived to the end of the experiment on day 12 p.c. and were euthanised on day 14 p.c. Together with the reduced clinical signs observed, this confirms the potential value of rVP2 as a component for development of serotype-specific subunit vaccines.

Mice challenged with wild-type BTV-4 or BTV-8 after immunisation with rVP2 of a heterologous serotype developed BTV RNAemia with CT values similar or 1 CT higher than those of the mock-immunised groups, indicating little or no cross-serotype protection (as was observed by comparison of clinical signs/mortality levels). However, when mice were immunised with rVP2s of BTV-4 or BTV-8 and then challenged with BTV-1RG_C7_, the mean CT values were reduced by up to 2.7 CT (Table S1, Figure 4). This increase in RNAemia (which was accompanied by more severe and fatal clinical signs) indicates increased BTV-1 replication in mice previously immunised with either of the two heterologous rVP2s, pointing to an ADE-like mechanism. Mice immunised with VP5 of BTV-10 and then challenged with BTV-4 or BTV-8 developed CT values (and severe clinical signs) similar to the mock-immunised mice after challenge with the same viruses (Figure 2 and Figure 3), indicating a lack of any significant protection. However, mice immunised with VP5 of BTV-10 and then challenged with BTV-1RG_C7_ (Figure 4) developed lower CT values on days 5 and 7 (20.17 and 19.9, respectively) compared to the mock-immunised mice challenged with the same virus (mean CT values of 22.7 and 28.1 on days 5 and 7, respectively). These lower CT values, along with more severe clinical signs starting on day 3 post-inoculation (observed clinical signs are defined in Table S2), including fatalities on day 7, suggest that an antibody-dependent enhancement mechanism involving an immune response to VP5 may be involved in increasing the replication and severity of infection with the attenuated BTV-1RG_C7_.

The CT values for blood samples collected on days 4–5 post-infection from mice challenged with BTV-1RG_C7_ (Figure 4, Table S1) were significantly different (*p* < 0.05—95% confidence—Table S5) for either the mock-immunised group (mild infection—Gp1) or the group of mice immunised with VP2 of BTV-1 (protected—Gp4), when compared to the groups immunised with VP2 of BTV-4 (Gp7), VP2 of BTV-8 (Gp10), or VP5 of BTV-10 (Gp13), which all showed severe and fatal infections.

## 4. Discussion

Previous studies involving immunisation of sheep with baculovirus expressed BTV virus-like particles (VLPs), showed high levels of neutralising antibodies and protection against challenge with the homologous serotype viruses (BTV-4, -10, -11, -13 and -17) [46], confirming the potential for the use of VLPs as vaccine components. There was also some indication of low levels of cross-serotype neutralising antibodies and partial protection (reduced clinical signs) against the heterologous serotypes, although no evidence of ADE was detected., The viruses involved have relatively closely related VP2 proteins, belonging to the same VP2 nucleotype-A [17] (Figure 1), which might help to explain a low level of cross neutralisation.

Our earlier studies of serological cross-reactions between the more distantly related BTV serotypes 4 and 8 [17,33] showed that IFNAR^(−/−)^ mice immunised with plant-expressed rVP2 proteins generated serotype-specific antibody responses against the homologous BTV serotype, although with relatively low neutralisation titres of 2–3 (the inverse of the serum dilution giving a 50% end-point in microtiter assays) and in ELISA using rVP2 as test antigens. In each case, the cross-serotype reactions were below detectable levels. Mice immunised with the rVP2 proteins were also protected against challenge with virulent strains of the homologous BTV serotype. However, these studies also indicated the possibility of ADE-type responses in mice immunised with rVP2 of one serotype going on to develop more severe clinical signs after challenge with the heterologous serotype.

The virulent wild-type strains of BTV used in these earlier studies caused very rapid fatalities in unprotected infected/challenged mice (within two to three days), giving only limited opportunities to observe any ADE-type responses that could potentially increase virus replication and severity, or further reduce survival times after heterologous serotype challenge. In the study reported here, we therefore included an attenuated, non-fatal clone of BTV-1RG_C7_ (generated by reverse genetics based on the genome sequence of the western reference strain of BTV-1 [35]) as a challenge strain, although the genetic basis and mechanism of attenuation, compared to the original BTV-1 reference strain, has not yet been determined.

Antisera previously raised in rabbits against rVP2 of BTV-1, BTV-4 and BTV-8 all reacted very strongly with the homologous proteins by ELISA [18]. The rabbit antiserum against rVP2 of BTV-8 also cross-reacted weakly by ELISA with the rVP2 proteins of both BTV-1 and BTV-4 [33], and the antiserum against rVP2 of BTV-4 showed a strong cross-reaction by ELISA with rVP2 of BTV-1 but did not cross-react with rVP2 of BTV-8 [33]. Rabbit antiserum against rVP2 of BTV-1 cross-reacted weakly by ELISA with rVP2 of BTV-4 but also failed to cross-react with rVP2 of BTV-8 [33]. Although strong reactions were also detected in each case by ELISA between the rVP2 proteins of these three serotypes and post-infected sheep reference sera for the homologous serotype, the only cross-reaction detected, between the BTV-8 antisera and rVP2 of BTV-4, was very weak [33].

The rabbit antisera raised against BTV rVP2 proteins and the ovine post-infected reference antisera against BTV-1, BTV-4 and BTV-8 all contained neutralising antibodies against the homologous serotype but failed to cross-neutralise either of the other serotypes used here [33].

The lowest CT values for BTV RNA in blood samples (highest RNAemia) observed in the study described here were in mice that were challenged with BTV1-RG_C7_ after heterologous-serotype immunisation with rVP2 of BTV-8 or rVP2 of BTV-4. This suggests the possibility an ADE mechanism(s) that enhances infection by this attenuated BTV-1. It is unclear if this is related specifically to the mechanism by which BTV-1RG_C7_ is attenuated (not yet determined), restoring virulence, or reflects serological relationships between the VP2 proteins of the different BTV strains involved.

Previous studies have ruled out a direct involvement of BTV VP5 as a neutralisation antigen [23,28], although it can help to determine virus serotype [21,22,39], possibly by imposing structural constraints on VP2. Mice immunised with rVP5 of BTV-10 and then challenged with the attenuated BTV-1 also provided evidence of enhanced infection. This suggests that antibody-dependent enhancement of infection/pathology is not restricted to interactions involving VP2 but could be mediated by interactions of any non-neutralising antibodies with the BTV outer-capsid layer. However, the restriction of these effects to BTV-1 in the present study, and as suggested by previous vaccination studies in calves vaccinated against BTV-8 and then challenged with BTV-9 [10], indicates that there may be some variation in the nature of any such response depending on the serotype specificity of the antibodies and the infecting/challenge strain involved.

Interactions between BTV virus particles and preformed but non-neutralising antibodies may enhance recognition, uptake and infection of dendritic macrophages [11] during the early stages of infection, increasing the speed of dissemination within the infected host prior to its own development of serotype-specific and protective neutralising antibodies. Early dissemination of infection could potentially enhance overall levels of virus replication, as well as increasing tissue damage. The interaction of BTV particles complexed with pre-existing antibodies could also lead to enhanced release of cytokines and endothelial cell death [10,11], resulting in increased severity of clinical signs. IFNAR^(−/−)^ mice have previously been used as a model for studies of the protection induced by experimental orbivirus vaccines or potential antiviral molecules [32,39,47,48,49,50,51]. The cross-protection that was observed in earlier studies after sequential vaccination (with live attenuated BTV vaccines) or infection with multiple BTV serotypes [52] may depend more on the cross-serotype reactivity of protective cell-mediated responses that have been demonstrated targeting BTV non-structural proteins [2], rather than any cross-serotype neutralising antibody responses, although we cannot rule out low-level cross neutralisation between strains containing closely related VP2 proteins [33]. Immunisation of sheep with baculovirus-expressed virus-like particles representing different BTV serotypes have indicated the possibility of a low-level cross-neutralising response that would likely mask any ADE-like effects. However, the serotypes that were tested in these earlier studies contained relatively closely related VP2 proteins belonging to VP2 Group A [17].

The severity of clinical signs that are observed after BTV vaccinations and infections appears likely to reflect an interplay of both antibody and cell-mediated responses in different individuals, depending on the relationships of different BTV strains and the previous vaccination or infection history of the host.

## 5. Conclusions

The severity of clinical signs and levels of BTV viraemia observed in ruminants in the field could be influenced by ADE-like mechanisms. These may be significant after vaccination campaigns using monovalent, serotype-specific vaccines (particularly if they are based primarily on VP2 and VP5 subunits) with potential to enhance the persistence of attenuated BTV vaccine strains that have previously been used in the field [31,53,54]. The use of inactivated vaccines (particularly in Europe) has inevitably led to widespread BTV-specific antibodies in the host animal populations that would be unlikely to neutralise other serotypes. However, ADE-like mechanisms could potentially increase the severity and/or facilitate the spread of subsequent outbreaks caused by these other viruses and might help to explain the detection of BTV-6 and BTV-11 vaccine strains in Europe [31,54].

The previous infection history and the resulting antibody specificity in individual animals or populations could influence the succession of different serotypes causing outbreaks in endemic areas, depending on the serological cross-reactivity of the outer-coat proteins of the different serotypes involved. Further studies in mice, sheep and cattle are needed to determine both the significance of serological relationships between BTV serotypes and the nature of any ADE mechanisms potentially involved. In view of the similarity of their structure, mode of replication and transmission, it is likely that any ADE mechanism affecting BTV could potentially also affect the members of other *Orbivirus* species and may even be relevant to other multi-serotype RNA viruses.

The results presented confirm the role of VP2 proteins and the serotype-specific antibodies generated against them in protective responses against the homologous BTV serotype, as well as indicating the potential of expressed VP2 proteins for use as components of subunit vaccines. However, they also indicate that it is important to consider the possibility of ADE responses when designing vaccination campaigns using serotype-specific inactivated or subunit vaccines. To prevent problems that could be caused by ADE, vaccines should include components that induce a more widely cross-serotype protective immune response, for example the more highly conserved BTV non-structural proteins NS1 and NS2, or structural protein VP7, which induce cross-reactive and protective T-cell mediated responses [2,55].

## Figures and Tables

**Table 1:** Groups of IFNAR^(−/−)^ mice immunized with rVP2 of BTV-1, BTV-4 or BTV-8 or with rVP5 of BTV-10, then challenged with live virus.

Group	Immunisation		Challenge
	Day 0 (Prime)	Day 14 (Boost)	Day 28
Group 1 (*n* = 5)	mock-immunised	mock-immunised	BTV-1RG_C7_ (control)
Group 2 (*n* = 5)	mock-immunised	mock-immunised	BTV-4RSArrrr/04 (control)
Group 3 (*n* = 5)	mock-immunised	mock-immunised	BTV-8RSArrrr/08 (control)
Group 4 (*n* = 5)	rVP2 of BTV-1	rVP2 of BTV-1	BTV-1RG_C7_ (homologous)
Group 5 (*n* = 5)	rVP2 of BTV-1	rVP2 of BTV-1	BTV-4RSArrrr/04 (heterologous)
Group 6 (*n* = 5)	rVP2 of BTV-1	rVP2 of BTV-1	BTV-8RSArrrr/08 (heterologous)
Group 7 (*n* = 5)	rVP2 of BTV-4	rVP2 of BTV-4	BTV-1RG_C7_ (heterologous)
Group 8 (*n* = 5)	rVP2 of BTV-4	rVP2 of BTV-4	BTV-4RSArrrr/04 (homologous)
Group 9 (*n* = 5)	rVP2 of BTV-4	rVP2 of BTV-4	BTV-8RSArrrr/08 (heterologous)
Group 10 (*n* = 5)	rVP2 of BTV-8	rVP2 of BTV-8	BTV-1RG_C7_ (heterologous)
Group 11 (*n* = 5)	rVP2 of BTV-8	rVP2 of BTV-8	BTV-4RSArrrr/04 (heterologous)
Group 12 (*n* = 5)	rVP2 of BTV-8	rVP2 of BTV-8	BTV-8RSArrrr/08 (homologous)
Group 13 (*n* = 5)	rVP5 of BTV-10	rVP5 of BTV-10	BTV-1RG_C7_ (heterologous)
Group 14 (*n* = 5)	rVP5 of BTV-10	rVP5 of BTV-10	BTV-4RSArrrr/04 (heterologous)
Group 15 (*n* = 5)	rVP5 of BTV-10	rVP5 of BTV-10	BTV-8RSArrrr/08 (heterologous)

## Data Availability

All data are presented in the main manuscript and supplementary material.

## Supplementary Materials

The following supporting information can be downloaded at: https://www.mdpi.com/article/10.3390/pathogens12040602/s1, Table S1: Summary of clinical signs and RNAemia in groups of mice mock-immunised with PBS or immunised with rVP2 or rVP5 of different serotypes and then challenged with BTV belonging to homologous or heterologous serotypes; Table S2: Observed mild or severe clinical signs in IFNAR^(−/−)^ mice infected with BTV; Table S3: The statistical significance of differences in the real-time RT-PCR results between groups of mice challenged with BTV-4RSArrrr/04; Table S4: The statistical significance of differences in the real-time RT-PCR results between groups of mice challenged with BTV-8RSArrrr/08; Table S5: The statistical significance of differences in the real-time RT-PCR results between groups of mice challenged with BTV-1RG_C7_.
