# Supplementary material for: Increased Clinical Signs and Mortality in IFNAR(−/−) Mice Immunised with the Bluetongue Virus Outer-Capsid Proteins VP2 or VP5, after Challenge with an Attenuated Heterologous Serotype
Source: Pathogens. 2023 Apr 15;12(4):602. doi: 10.3390/pathogens12040602 (PMC10141489; doi:10.3390/pathogens12040602)
Supplement: Supplementary file 1 [file pathogens-12-00602-s001.zip › pathogens-2273720-supplementary.pdf]

Table S1. Summary of clinical signs and RNAemia in groups of mice mock immunised with PBS, or immunised with rVP2 or rVP5 of different serotypes, then challenged with BTV belonging to homologous or heterologous serotypes

**Group (immunogen / challenge virus)**	**Day 4-Day 5**	**Day 7**	**Day 12**
**Group1** (mock / BTV-1RG$_{C7}$)	Mild Clinical signs, all mice alive **CT=**22.17; 21.53; 24.10; 22.85; 21.87, **Mean=** 22.7	Mild Clinical signs, all mice alive: **CT=**28.12; 27.08; 29.55; 25.83; 29.47, **Mean=** 28.01	All mice recovered: **CT=** 39.34; 38.15; 37.06; 39.14; 38.66, **Mean=** 38.47
**Group2** (mock / BTV-4RSArrrr/04 control)	Severe clinical signs (day 4), all mice died on day 4: **CT=**20.63; 21.67; 22.21; 23.41; 22.64, **Mean=**22.11		
**Group3** (mock / BTV-8RSArrrr/08 control)	Severe clinical signs (day 4), 4 mice died on day 4 and one died on day 5 **CT=**22.45; 21.27; 23.45; 23.11; 21.64, **Mean=**22.38		
**Group4** (VP2 BTV-1 / BTV-1RG$_{C7}$)	No clinical signs, all mice alive **CT=**26.91; 28.37; 26.82; 29.73; 27.10, **Mean=** 27.78	No clinical signs, all mice alive: **CT=**29.47; 29.9; 29.24; 30.65; 30.01, **Mean= 29.87**	All mice alive : **CT=**39.61; 38.45; 39.26; 37.94; 40.12, **Mean=** 39.07
**Group5** (VP2 BTV-1 / BTV-4RSArrrr/04)	Severe clinical signs (day 4), All mice died on day 4 **CT=**21.63; 22.84; 24.13; 23.77; 24.75, **Mean=** 23.42		
**Group6** (VP2 BTV-1 / BTV-8RSArrrr/08)	Severe clinical signs (day 4), all mice died day 4 **CT=**23.97; 23.84; 21.61; 22.47; 24.98, **Mean=** 23.37		
**Group7** (VP2 BTV-4 / BTV-1RG$_{C7}$)	Severe clinical signs (day4-day5), one mouse died on day 4 and 4 mice died on day 5 **CT=**20.42; 19. 47; 20.98; 20.59; 18.61, **Mean=** 20.01		
**Group8** (VP2 BTV-4 / BTV-4RSArrrr/04)	No clinical signs all alive **CT=**26.43; 27.61; 27.73; 30.13; 28.64, **Mean=** 28.10	No clinical signs, all mice alive: **CT=**30.85; 30.37; 34.79; 33.44; 35.31, **Mean=** 32.95	All mice alive: **CT=**37.10; 36.67; 38.06; 40.72; 39.09, **Mean=** 37.72
**Group9** (VP2 BTV-4 / BTV-8RSArrrr/08)	Severe clinical signs, all mice died on day 4 **CT=**22.13; 20.86; 20.41; 21.35; 20.72, **Mean=**		
**Group10** (VP2 BTV-8 / BTV-1RG$_{C7}$)	Mild/severe clinical signs (day 4-day 5), all mice died day 5: **CT=**18.52; 20.55; 21.69; 19.38; 20.69, **Mean=** 20.16		
**Group11** (VP2 BTV-8 / BTV-4RSArrrr/04)	Severe clinical signs (day 4), all mice died day 4 **CT=**23.98; 21.17; 22.06; 23.11 21.53, **Mean=** 22.37		
**Group12** (VP2 BTV-8 / BTV-8RSArrrr/08)	Mild Clinical signs, all mice alive **CT=**29.84; 28.29; 30.01; 27.64; 27.12, **Mean=** 28.6	No clinical signs, all mice alive: **CT=**33.68; 34.12; 35.52; 32.19; 31.09, **Mean=** 33.32	All mice alive: **CT=**39.12; 40.01; 40.10; 39.82; 40.12, **Mean=** 39.83
**Group13** (VP5 BTV-10 / BTV-1RG$_{C7}$)	Mild/sever clinical signs (day 5) **CT=**19.97; 20.7; 19.57; 21.35; 19.26, **Mean=** 20.17	Severe clinical signs, all mice died day 7: **CT=**19.62; 21.01; 18.91; 20.40; 19.56, **Mean=** 19.9	
**Group14** (VP5 BTV-10 / BTV-4RSArrrr/04)	Severe clinical signs (day 4), all mice died on day 4. **CT=**22.53; 22.40; 23.99; 21.71; 21.94, **Mean=** 22.51		
**Group15** (VP5 BTV-10 / BTV-8RSArrrr/08)	Severe clinical signs (day 4), all mice died on day 4 **CT=**20.10; 23.05; 24.1; 21.14; 22.56, **Mean=** 22.19		

Table S2: Observed mild or severe clinical signs in IFNAR$^{(-/-)}$ mice infected with BTV.

Observed mild clinical signs	Observed severe clinical signs
Scruffy fur	Scruffy fur
Mild lacrimation	Profuse lacrimation
Eye lids intermittently shut	Eye lids permanently shut
Reduced mobility	Sticky secretions from the eyes
	Prostration
	Dehydration

The mice that developed mild clinical signs, recovered and survived, while mice that developed severe clinical signs had all died by day 4 to 7 post challenge.

Table S3: The statistical significance of differences in the real-time RT-PCR results between groups of mice challenged with BTV-4RSArrrr/04.

Compared groups	Tukey HSD calculated p-value	Significance
Gp2 / Gp5	0.3997127	N.S.
Gp2 / Gp8	0.0010053	** p<0.01
Gp2 / Gp11	0.8999947	N.S.
Gp2 / Gp14	0.8999947	N.S.
Gp5 / Gp8	0.0010053	** p<0.01
Gp5 / Gp11	0.5937424	N.S.
Gp5 / Gp14	0.7005442	N.S.
Gp8 / Gp11	0.0010053	** p<0.01
Gp8 / Gp14	0.0010053	** p<0.01
Gp11 / Gp14	0.8999947	N.S.

Analysis of variance (ANOVA) using Tukey's test with HSD for the real-time RT-PCR Ct values of RNA extract from blood samples collected on day 4-5 post-challenge with BTV-4RSArrrr/04. **N.S.:** not significant

Gp2: mock-immunised
Gp5: immunised with VP2 BTV-1
Gp8: immunised with VP2 BTV-4
Gp11: immunised with VP2 BTV-8
Gp14: immunised with VP5 BTV-10

Table S4: The statistical significance of differences in the real-time RT-PCR results between groups of mice challenged with BTV-8RSArrrr/08.

Compared groups	Tukey HSD calculated p-value	Significance
Gp3 / Gp6	0.6753846	N.S.
Gp3 / Gp9	0.4620797	N.S.
Gp3 / Gp 12	0.0010053	** p<0.01
Gp3 / Gp15	0.8999947	N.S.
Gp6 / Gp9	0.0502538	N.S.
Gp6 / Gp12	0.0010053	** p<0.01
Gp6 / Gp15	0.5381951	N.S.
Gp9 / Gp12	0.0010053	** p<0.01
Gp9 / Gp15	0.6004254	N.S.
Gp12 / Gp15	0.0010053	** p<0.01

Analysis of variance (ANOVA) using Tukey's test with HSD for the real-time RT-PCR Ct values of RNA extract from blood samples collected on day 4-5 post-challenge with BTV-8RSArrrr/08. **N.S:** not significant

Gp3: mock-immunised
Gp6: immunised with VP2 BTV-1
Gp9: immunised withVP2 BTV-4
Gp12: immunised with VP2 BTV-8
Gp15: immunised with VP5 BTV-10

Table S5: The statistical significance of differences in the real-time RT-PCR results between groups of mice challenged with BTV-1RG$_{C7}$.

Compared groups	Tukey HSD calculated p-value	Significance
Gp1 / Gp4	0.0010053	** p<0.01
Gp1 / Gp7	0.0119114	* p<0.05
Gp1 / Gp10	0.0194239	* p<0.05
Gp1 / Gp13	0.0196736	* p<0.05
Gp1 / Gp13*	0.0065790	** p<0.01
GP4 / Gp7	0.0010053	** p<0.01
Gp4 / Gp10	0.0010053	** p<0.01
Gp4 / Gp13	0.0010053	** p<0.01
Gp4 / Gp13*	0.0010053	** p<0.01
Gp7 / Gp10	0.8999947	N.S.
Gp7 / Gp13	0.8999947	N.S.
Gp7 / Gp13*	0.8999947	N.S.
Gp10 / Gp13	0.8999947	N.S.
Gp13 / Gp13*	0.8999947	N.S.

Analysis of variance (ANOVA) using Tukey's test with HSD for the real-time RT-PCR Ct values of RNA extract from blood samples collected on day 4-5 post-challenge with BTV-1RG$_{C7}$.
* samples collected on day 7 from mice of group 13 before they died. **N.S:** not significant

Gp1: mock-immunised
Gp4: immunised with VP2 BTV-1
Gp7: immunised with VP2 BTV-4
Gp10: immunised with VP2 BTV-8
Gp13: immunised with VP5 BTV-10
